# Second Class Postal Rates

**Published:** 1912-08

**Authors:** 


### Second Class Postal Rates

IT has been a surprise to us that the Post Office, after having shown a surplus for the first time in its history, should have been so insistant on the raising of second class rates. Mr. Wilmer Atkinson, Editor of the *Farm Journal,* explains this by the statement that over seven millions of expenses have been carried over into the present fiscal year. Another inexplicable fact in the controversy over second class rates is the obstinate contention that the post office loses something like 7 cents a pound on cent-a-pound matter, when its aggregate, per capita and net profits have steadily increased as second-class mail matter has increased.

The practical point for our readers to consider is this: Do you want to pay more for all of your periodical reading matter or would you rather have the government make good a slight deficit in the Post Office by an indirect tax or by foregoing a battle ship or in some other indirect way? It makes comparatively little difference to the BUFFALO MEDICAL JOURNAL or to other monthlies of moderate weight but, in the long run, a postal surplus will cost the people dearly.

